# Tris(2,2′-bipyridine-κ^2^ *N*,*N*′)cadmium(II) bis­(perchlorate) hemihydrate

**DOI:** 10.1107/S1600536808036593

**Published:** 2008-12-03

**Authors:** Weiguang Zhang, Zhengjing Jiang, Lude Lu

**Affiliations:** a Key Laboratory for Soft Chemistry and Functional Materials of the Ministry of Education, Nanjing University of Science and Technology, Nanjing 210094, People’s Republic of China

## Abstract

The asymmetric unit of the title compound, [Cd(C_10_H_8_N_2_)_3_](ClO_4_)_2_·0.5H_2_O, consists of one complex [Cd(bpy)_3_]^2+^ cation (bpy = 2,2′-bipyridine), two perchlorate anions and one water molecule with half-occupancy. The central cadmium(II) ion is bound to six N atoms from three bpy ligands in a distorted octa­hedral coordination, with Cd—N bond distances ranging from 2.304 (3) to 2.395 (2) Å.

## Related literature

For applications of metal complexes of 2,2′-bipyridine and its derivatives, see: Kuang *et al.* (2006 ▶). For cadmium complexes, see: Kundu *et al.* (2005 ▶); Ranjbar *et al.* (2007 ▶); Shi *et al.* (2006 ▶); Zheng *et al.* (2005 ▶).
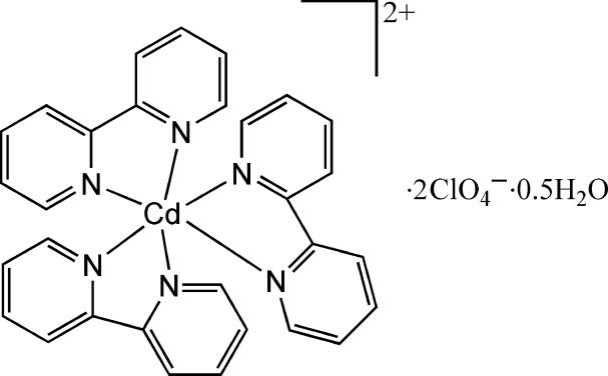


## Experimental

### 

#### Crystal data

[Cd(C_10_H_8_N_2_)_3_](ClO_4_)_2_·0.5H_2_O
*M* *_r_* = 788.87
Triclinic, 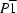

*a* = 8.1704 (3) Å
*b* = 11.0282 (5) Å
*c* = 18.3875 (7) Å
α = 104.631 (1)°
β = 92.652 (1)°
γ = 100.520 (1)°
*V* = 1568.68 (11) Å^3^
*Z* = 2
Mo *K*α radiation
μ = 0.93 mm^−1^
*T* = 173 (2) K
0.20 × 0.16 × 0.12 mm

#### Data collection

Bruker SMART CCD area-detector diffractometer
Absorption correction: multi-scan (*SADABS*; Bruker, 2000 ▶) *T*_min_ = 0.82, *T*_max_ = 0.90
19563 measured reflections
6067 independent reflections
5114 reflections with *I* > 2σ(*I*)
*R*_int_ = 0.037

#### Refinement

*R*[*F* ^2^ > 2σ(*F* ^2^)] = 0.034
*wR*(*F* ^2^) = 0.107
*S* = 1.01
6067 reflections
434 parameters
H-atom parameters constrained
Δρ_max_ = 0.44 e Å^−3^
Δρ_min_ = −0.54 e Å^−3^

### 

Data collection: *SMART* (Bruker, 2000 ▶); cell refinement: *SAINT* (Bruker, 2000 ▶); data reduction: *SAINT*; program(s) used to solve structure: *SHELXS97* (Sheldrick, 2008 ▶); program(s) used to refine structure: *SHELXL97* (Sheldrick, 2008 ▶); molecular graphics: *SHELXTL* (Sheldrick, 2008 ▶); software used to prepare material for publication: *SHELXTL*.

## Supplementary Material

Crystal structure: contains datablocks I, global. DOI: 10.1107/S1600536808036593/hg2440sup1.cif

Structure factors: contains datablocks I. DOI: 10.1107/S1600536808036593/hg2440Isup2.hkl

Additional supplementary materials:  crystallographic information; 3D view; checkCIF report

## Figures and Tables

**Table 1:** Selected geometric parameters (Å, °)

Cd1—N4	2.304 (3)
Cd1—N2	2.312 (3)
Cd1—N6	2.330 (3)
Cd1—N3	2.329 (3)
Cd1—N5	2.383 (3)
Cd1—N1	2.395 (2)

**Fig. 1.**
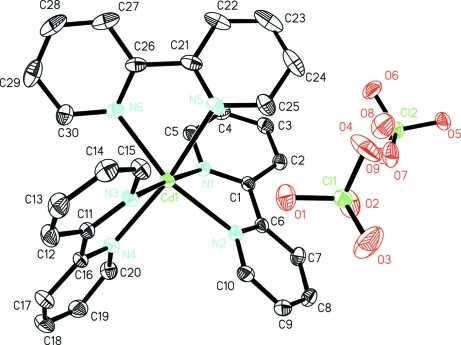
View of the title compound. Displacement ellipsoids for non-H atoms are drawn at the 30% probability level.

## supplementary crystallographic information

### Comment

Metal complexes of 2,2'-bipyridine and its derivatives have several applications, for example, in dye-sensitized solar cells (Kuang *et al.*, 2006). Among these reported complexes, only a few structurally characterized examples are of cadmium ion (Kundu *et al.*, 2005; Ranjbar *et al.*, 2007; Shi *et al.*, 2006; Zheng *et al.*, 2005). For these cadmium complexes, the coordination scheme gives rise to monomeric species. Here we report a new monomeric cadmium(II) complex, *viz.* the title compound, [Cd(C_10_H_8_N_2_)_3_](ClO_4_)_2_.0.5H_2_O (I).

The structure of (I) consists of monomeric [Cd(bpy)_3_]_2+_ cations (bpy = 2,2'-bipyridine) and perchlorate anions and non-coordinating water molecules. The cadmium(II) ion is bound to six nitrogen atoms from three bpy ligands, giving a distorted CdN_6_ octahedral geometry. Of the six Cd—N bond distances ranging from 2.304 (3) to 2.395 (2) Å, the two *cis* bonds, Cd1—N1 and Cd1—N5, are longer than the other four bonds. The three 2,2'-bipyridine ligands are bent, with dihedral angles between the mean planes of two pyridine rings of the same bipyridine ligand ranging from 11.0 (2) to 27.6 (2)°.

### Experimental

The title complex was obtained as light red crystals by the hydrothermal reaction of Cd(ClO_4_)_2_.6H_2_O (0.10 mmol) and 2,2'-bipyridine (0.10 mmol) in water (7 ml) at 160°C for 48 hr.

### Refinement

H atoms bonded to O atoms of water molecules were located in a difference map and refined with O—H bonds = 0.85 Å, and with *U*_iso_(H) = 1.2*U*_eq_(O). Other H atoms were positioned geometrically and refined using a riding model, with C—H bonds = 0.95 Å and with *U*_iso_ (H) = 1.2*U*_eq_ (C).

### Crystal data

**Table:**

[Cd(C_10_H_8_N_2_)_3_](ClO_4_)_2_·0.5H_2_O	*Z* = 2
*M**_r_* = 788.87	*F*(000) = 794
Triclinic, *P*1	*D*_x_ = 1.670 Mg m^−^^3^
Hall symbol: -P 1	Mo *K*α radiation, λ = 0.71073 Å
*a* = 8.1704 (3) Å	Cell parameters from 5350 reflections
*b* = 11.0282 (5) Å	θ = 2.3–24.2°
*c* = 18.3875 (7) Å	µ = 0.93 mm^−^^1^
α = 104.631 (1)°	*T* = 173 K
β = 92.652 (1)°	Block, light red
γ = 100.520 (1)°	0.20 × 0.16 × 0.12 mm
*V* = 1568.68 (11) Å^3^	

### Data collection

**Table:**

Bruker SMART CCD area-detector diffractometer	6067 independent reflections
Radiation source: fine-focus sealed tube	5114 reflections with *I* > 2σ(*I*)
graphite	*R*_int_ = 0.037
φ and ω scans	θ_max_ = 26.0°, θ_min_ = 1.2°
Absorption correction: multi-scan (*SADABS*; Bruker, 2000)	*h* = −9→10
*T*_min_ = 0.82, *T*_max_ = 0.90	*k* = −13→13
19563 measured reflections	*l* = −22→22

### Refinement

**Table:**

Refinement on *F*^2^	Primary atom site location: structure-invariant direct methods
Least-squares matrix: full	Secondary atom site location: difference Fourier map
*R*[*F*^2^ > 2σ(*F*^2^)] = 0.034	Hydrogen site location: inferred from neighbouring sites
*wR*(*F*^2^) = 0.107	H-atom parameters constrained
*S* = 1.01	*w* = 1/[σ^2^(*F*_o_^2^) + (0.0692*P*)^2^] where *P* = (*F*_o_^2^ + 2*F*_c_^2^)/3
6067 reflections	(Δ/σ)_max_ = 0.001
434 parameters	Δρ_max_ = 0.44 e Å^−^^3^
0 restraints	Δρ_min_ = −0.54 e Å^−^^3^

### Special details

**Table:**

Geometry. All e.s.d.'s (except the e.s.d. in the dihedral angle between two l.s. planes) are estimated using the full covariance matrix. The cell e.s.d.'s are taken into account individually in the estimation of e.s.d.'s in distances, angles and torsion angles; correlations between e.s.d.'s in cell parameters are only used when they are defined by crystal symmetry. An approximate (isotropic) treatment of cell e.s.d.'s is used for estimating e.s.d.'s involving l.s. planes.
Refinement. Refinement of *F*^2^ against ALL reflections. The weighted *R*-factor *wR* and goodness of fit *S* are based on *F*^2^, conventional *R*-factors *R* are based on *F*, with *F* set to zero for negative *F*^2^. The threshold expression of *F*^2^ > σ(*F*^2^) is used only for calculating *R*-factors(gt) *etc*. and is not relevant to the choice of reflections for refinement. *R*-factors based on *F*^2^ are statistically about twice as large as those based on *F*, and *R*- factors based on ALL data will be even larger.

### Fractional atomic coordinates and isotropic or equivalent isotropic displacement parameters (Å^2^)

**Table:**

	*x*	*y*	*z*	*U*_iso_*/*U*_eq_	Occ. (<1)
Cd1	0.49907 (3)	0.73554 (2)	0.243298 (12)	0.03097 (10)	
N1	0.6598 (3)	0.6830 (2)	0.33902 (14)	0.0294 (6)	
N2	0.6508 (3)	0.5860 (2)	0.18631 (14)	0.0301 (6)	
N3	0.3534 (3)	0.7832 (3)	0.14562 (14)	0.0322 (6)	
N4	0.6676 (3)	0.8972 (3)	0.20701 (14)	0.0317 (6)	
N5	0.2893 (3)	0.5889 (3)	0.28060 (15)	0.0343 (6)	
N6	0.3367 (3)	0.8481 (3)	0.32562 (14)	0.0336 (6)	
C1	0.7042 (3)	0.5700 (3)	0.31281 (16)	0.0275 (6)	
C2	0.7179 (4)	0.4891 (3)	0.35877 (18)	0.0348 (7)	
H2	0.7476	0.4086	0.3390	0.042*	
C3	0.6876 (4)	0.5275 (3)	0.43375 (19)	0.0402 (8)	
H3	0.6949	0.4733	0.4661	0.048*	
C4	0.6469 (4)	0.6444 (4)	0.46057 (19)	0.0399 (8)	
H4	0.6284	0.6735	0.5122	0.048*	
C5	0.6330 (4)	0.7198 (3)	0.41210 (17)	0.0336 (7)	
H5	0.6034	0.8006	0.4310	0.040*	
C6	0.7320 (4)	0.5340 (3)	0.23180 (17)	0.0280 (6)	
C7	0.8391 (4)	0.4527 (3)	0.20353 (19)	0.0373 (8)	
H7	0.8951	0.4154	0.2360	0.045*	
C8	0.8633 (4)	0.4266 (3)	0.1278 (2)	0.0411 (8)	
H8	0.9369	0.3718	0.1077	0.049*	
C9	0.7789 (4)	0.4813 (3)	0.08131 (19)	0.0398 (8)	
H9	0.7934	0.4651	0.0290	0.048*	
C10	0.6737 (4)	0.5596 (3)	0.11315 (18)	0.0352 (7)	
H10	0.6146	0.5965	0.0815	0.042*	
C11	0.4207 (4)	0.8910 (3)	0.12777 (17)	0.0312 (7)	
C12	0.3271 (5)	0.9441 (4)	0.0842 (2)	0.0444 (9)	
H12	0.3754	1.0210	0.0723	0.053*	
C13	0.1647 (5)	0.8857 (4)	0.0582 (2)	0.0491 (9)	
H13	0.0998	0.9217	0.0282	0.059*	
C14	0.0970 (4)	0.7748 (4)	0.07580 (19)	0.0447 (9)	
H14	−0.0149	0.7320	0.0580	0.054*	
C15	0.1953 (4)	0.7269 (3)	0.12003 (19)	0.0414 (8)	
H15	0.1485	0.6505	0.1329	0.050*	
C16	0.5991 (4)	0.9476 (3)	0.15684 (17)	0.0330 (7)	
C17	0.6922 (5)	1.0456 (4)	0.1331 (2)	0.0464 (9)	
H17	0.6415	1.0839	0.0996	0.056*	
C18	0.8591 (5)	1.0872 (4)	0.1585 (2)	0.0555 (11)	
H18	0.9249	1.1541	0.1423	0.067*	
C19	0.9301 (4)	1.0308 (4)	0.2077 (2)	0.0468 (9)	
H19	1.0460	1.0556	0.2244	0.056*	
C20	0.8297 (4)	0.9392 (3)	0.23142 (19)	0.0409 (8)	
H20	0.8769	0.9030	0.2671	0.049*	
C21	0.2114 (4)	0.6420 (3)	0.34008 (17)	0.0365 (8)	
C22	0.1362 (4)	0.5700 (4)	0.3857 (2)	0.0515 (11)	
H22	0.0852	0.6096	0.4283	0.062*	
C23	0.1353 (5)	0.4418 (5)	0.3693 (2)	0.0656 (14)	
H23	0.0832	0.3916	0.4001	0.079*	
C24	0.2102 (5)	0.3873 (4)	0.3083 (3)	0.0612 (13)	
H24	0.2092	0.2981	0.2950	0.073*	
C25	0.2881 (4)	0.4648 (3)	0.2658 (2)	0.0450 (9)	
H25	0.3432	0.4270	0.2241	0.054*	
C26	0.2157 (4)	0.7800 (3)	0.35443 (17)	0.0357 (8)	
C27	0.0983 (4)	0.8389 (5)	0.3950 (2)	0.0543 (11)	
H27	0.0124	0.7901	0.4149	0.065*	
C28	0.1078 (5)	0.9681 (5)	0.4058 (2)	0.0625 (13)	
H28	0.0298	1.0098	0.4339	0.075*	
C29	0.2321 (6)	1.0361 (4)	0.3754 (2)	0.0587 (12)	
H29	0.2398	1.1251	0.3814	0.070*	
C30	0.3449 (5)	0.9735 (4)	0.3362 (2)	0.0434 (9)	
H30	0.4317	1.0209	0.3159	0.052*	
Cl1	0.30915 (10)	0.28250 (8)	0.06302 (4)	0.03444 (19)	
O1	0.2886 (4)	0.4107 (3)	0.07130 (16)	0.0558 (7)	
O2	0.4439 (3)	0.2792 (3)	0.11403 (16)	0.0599 (8)	
O3	0.3387 (6)	0.2297 (4)	−0.01150 (18)	0.1051 (14)	
O4	0.1617 (4)	0.2129 (3)	0.0806 (2)	0.0909 (13)	
Cl2	0.75289 (11)	0.16580 (8)	0.41261 (5)	0.0389 (2)	
O5	0.7279 (3)	0.0316 (2)	0.40528 (15)	0.0488 (6)	
O6	0.6449 (3)	0.2223 (3)	0.46332 (17)	0.0605 (8)	
O7	0.9241 (3)	0.2243 (3)	0.44150 (15)	0.0558 (7)	
O8	0.7206 (4)	0.1863 (2)	0.34000 (15)	0.0639 (8)	
O9	0.4144 (9)	0.1855 (6)	0.2510 (3)	0.0752 (19)	0.50
H9A	0.4323	0.2135	0.2125	0.090*	0.50
H9B	0.5080	0.1881	0.2741	0.090*	0.50

### Atomic displacement parameters (Å^2^)

**Table:**

	*U*^11^	*U*^22^	*U*^33^	*U*^12^	*U*^13^	*U*^23^
Cd1	0.03118 (15)	0.03525 (17)	0.03679 (15)	0.01384 (10)	0.01081 (10)	0.02177 (11)
N1	0.0266 (13)	0.0319 (15)	0.0321 (13)	0.0070 (11)	0.0015 (10)	0.0123 (11)
N2	0.0314 (14)	0.0286 (14)	0.0346 (14)	0.0091 (11)	0.0050 (11)	0.0136 (11)
N3	0.0347 (14)	0.0329 (15)	0.0339 (14)	0.0088 (12)	0.0074 (11)	0.0158 (12)
N4	0.0332 (14)	0.0316 (15)	0.0331 (14)	0.0070 (11)	0.0079 (11)	0.0123 (12)
N5	0.0298 (14)	0.0376 (17)	0.0343 (14)	−0.0015 (12)	−0.0016 (11)	0.0144 (12)
N6	0.0276 (13)	0.0422 (17)	0.0334 (14)	0.0115 (12)	0.0054 (11)	0.0108 (12)
C1	0.0245 (15)	0.0268 (16)	0.0332 (15)	0.0059 (12)	0.0013 (12)	0.0115 (13)
C2	0.0365 (17)	0.0321 (18)	0.0413 (18)	0.0124 (14)	0.0049 (14)	0.0155 (15)
C3	0.044 (2)	0.044 (2)	0.0403 (18)	0.0098 (16)	0.0021 (15)	0.0245 (16)
C4	0.0398 (19)	0.052 (2)	0.0304 (16)	0.0107 (16)	0.0043 (14)	0.0142 (16)
C5	0.0330 (17)	0.0354 (19)	0.0342 (16)	0.0093 (14)	0.0040 (13)	0.0104 (14)
C6	0.0260 (15)	0.0258 (16)	0.0349 (16)	0.0062 (12)	0.0033 (12)	0.0120 (13)
C7	0.0389 (18)	0.0336 (18)	0.0431 (18)	0.0155 (15)	0.0008 (14)	0.0117 (15)
C8	0.0415 (19)	0.0353 (19)	0.048 (2)	0.0167 (15)	0.0110 (15)	0.0067 (16)
C9	0.048 (2)	0.038 (2)	0.0345 (17)	0.0082 (16)	0.0124 (15)	0.0109 (15)
C10	0.0399 (18)	0.0368 (19)	0.0338 (17)	0.0095 (15)	0.0081 (14)	0.0163 (14)
C11	0.0402 (18)	0.0288 (17)	0.0295 (15)	0.0120 (14)	0.0088 (13)	0.0123 (13)
C12	0.053 (2)	0.042 (2)	0.047 (2)	0.0158 (17)	0.0025 (17)	0.0229 (17)
C13	0.055 (2)	0.054 (2)	0.049 (2)	0.0261 (19)	−0.0013 (17)	0.0233 (19)
C14	0.0367 (19)	0.056 (2)	0.0426 (19)	0.0150 (17)	−0.0029 (15)	0.0124 (17)
C15	0.0425 (19)	0.039 (2)	0.0441 (19)	0.0038 (15)	0.0016 (15)	0.0179 (16)
C16	0.0432 (18)	0.0288 (17)	0.0329 (16)	0.0102 (14)	0.0127 (14)	0.0153 (14)
C17	0.054 (2)	0.044 (2)	0.048 (2)	0.0061 (17)	0.0139 (17)	0.0257 (17)
C18	0.059 (3)	0.042 (2)	0.065 (3)	−0.0041 (19)	0.023 (2)	0.020 (2)
C19	0.0372 (19)	0.045 (2)	0.053 (2)	0.0009 (16)	0.0099 (16)	0.0086 (18)
C20	0.041 (2)	0.038 (2)	0.0427 (19)	0.0066 (16)	0.0029 (15)	0.0106 (16)
C21	0.0248 (16)	0.053 (2)	0.0295 (16)	−0.0026 (14)	−0.0019 (12)	0.0162 (15)
C22	0.0375 (19)	0.074 (3)	0.0388 (19)	−0.0165 (19)	−0.0037 (15)	0.0289 (19)
C23	0.050 (2)	0.088 (4)	0.054 (3)	−0.028 (2)	−0.015 (2)	0.044 (3)
C24	0.061 (3)	0.047 (2)	0.071 (3)	−0.018 (2)	−0.025 (2)	0.035 (2)
C25	0.043 (2)	0.039 (2)	0.051 (2)	−0.0024 (16)	−0.0087 (16)	0.0181 (17)
C26	0.0251 (16)	0.056 (2)	0.0271 (15)	0.0074 (15)	0.0023 (12)	0.0129 (15)
C27	0.0343 (19)	0.097 (4)	0.0361 (19)	0.026 (2)	0.0081 (15)	0.016 (2)
C28	0.053 (3)	0.103 (4)	0.041 (2)	0.050 (3)	0.0073 (18)	0.011 (2)
C29	0.075 (3)	0.057 (3)	0.047 (2)	0.041 (2)	−0.010 (2)	0.003 (2)
C30	0.043 (2)	0.040 (2)	0.048 (2)	0.0144 (16)	−0.0003 (16)	0.0095 (17)
Cl1	0.0324 (4)	0.0375 (5)	0.0363 (4)	0.0062 (3)	0.0032 (3)	0.0155 (3)
O1	0.0639 (18)	0.0448 (16)	0.0671 (18)	0.0208 (14)	0.0003 (14)	0.0238 (14)
O2	0.0509 (16)	0.0511 (17)	0.080 (2)	0.0109 (13)	−0.0190 (14)	0.0257 (15)
O3	0.184 (4)	0.103 (3)	0.0511 (19)	0.082 (3)	0.038 (2)	0.0193 (19)
O4	0.0405 (16)	0.090 (3)	0.167 (4)	0.0002 (16)	0.0150 (19)	0.087 (3)
Cl2	0.0446 (5)	0.0342 (5)	0.0418 (4)	0.0122 (4)	0.0064 (4)	0.0137 (4)
O5	0.0504 (15)	0.0356 (14)	0.0681 (17)	0.0119 (11)	0.0114 (13)	0.0242 (13)
O6	0.0511 (16)	0.0616 (18)	0.0702 (19)	0.0283 (14)	0.0178 (14)	0.0060 (15)
O7	0.0423 (14)	0.0619 (18)	0.0617 (17)	−0.0040 (13)	0.0091 (12)	0.0235 (14)
O8	0.109 (2)	0.0407 (16)	0.0478 (15)	0.0232 (16)	−0.0063 (15)	0.0195 (13)
O9	0.105 (5)	0.075 (4)	0.066 (4)	0.045 (4)	0.018 (3)	0.036 (3)

### Geometric parameters (Å, °)

**Table:**

Cd1—N4	2.304 (3)	C13—H13	0.9500
Cd1—N2	2.312 (3)	C14—C15	1.377 (5)
Cd1—N6	2.330 (3)	C14—H14	0.9500
Cd1—N3	2.329 (3)	C15—H15	0.9500
Cd1—N5	2.383 (3)	C16—C17	1.380 (5)
Cd1—N1	2.395 (2)	C17—C18	1.375 (5)
N1—C1	1.340 (4)	C17—H17	0.9500
N1—C5	1.343 (4)	C18—C19	1.382 (6)
N2—C10	1.332 (4)	C18—H18	0.9500
N2—C6	1.341 (4)	C19—C20	1.356 (5)
N3—C15	1.334 (4)	C19—H19	0.9500
N3—C11	1.341 (4)	C20—H20	0.9500
N4—C20	1.337 (4)	C21—C22	1.384 (5)
N4—C16	1.341 (4)	C21—C26	1.471 (5)
N5—C25	1.325 (4)	C22—C23	1.368 (6)
N5—C21	1.349 (4)	C22—H22	0.9500
N6—C30	1.336 (4)	C23—C24	1.362 (7)
N6—C26	1.342 (4)	C23—H23	0.9500
C1—C2	1.390 (4)	C24—C25	1.389 (5)
C1—C6	1.481 (4)	C24—H24	0.9500
C2—C3	1.384 (5)	C25—H25	0.9500
C2—H2	0.9500	C26—C27	1.395 (5)
C3—C4	1.367 (5)	C27—C28	1.375 (6)
C3—H3	0.9500	C27—H27	0.9500
C4—C5	1.378 (5)	C28—C29	1.376 (6)
C4—H4	0.9500	C28—H28	0.9500
C5—H5	0.9500	C29—C30	1.374 (5)
C6—C7	1.391 (4)	C29—H29	0.9500
C7—C8	1.380 (5)	C30—H30	0.9500
C7—H7	0.9500	Cl1—O3	1.398 (3)
C8—C9	1.389 (5)	Cl1—O4	1.404 (3)
C8—H8	0.9500	Cl1—O2	1.425 (2)
C9—C10	1.374 (5)	Cl1—O1	1.425 (3)
C9—H9	0.9500	Cl2—O6	1.422 (3)
C10—H10	0.9500	Cl2—O5	1.427 (3)
C11—C12	1.384 (4)	Cl2—O8	1.430 (3)
C11—C16	1.492 (4)	Cl2—O7	1.444 (3)
C12—C13	1.370 (5)	O9—H9A	0.8500
C12—H12	0.9500	O9—H9B	0.8500
C13—C14	1.370 (5)		
			
N4—Cd1—N2	92.41 (9)	C11—C12—H12	120.0
N4—Cd1—N6	101.98 (10)	C12—C13—C14	119.3 (3)
N2—Cd1—N6	162.88 (10)	C12—C13—H13	120.4
N4—Cd1—N3	71.23 (9)	C14—C13—H13	120.4
N2—Cd1—N3	106.18 (9)	C13—C14—C15	118.3 (3)
N6—Cd1—N3	87.41 (9)	C13—C14—H14	120.8
N4—Cd1—N5	170.37 (9)	C15—C14—H14	120.8
N2—Cd1—N5	96.17 (10)	N3—C15—C14	122.8 (3)
N6—Cd1—N5	70.34 (10)	N3—C15—H15	118.6
N3—Cd1—N5	102.03 (9)	C14—C15—H15	118.6
N4—Cd1—N1	107.32 (9)	N4—C16—C17	120.7 (3)
N2—Cd1—N1	70.96 (9)	N4—C16—C11	116.7 (3)
N6—Cd1—N1	95.64 (9)	C17—C16—C11	122.6 (3)
N3—Cd1—N1	176.86 (8)	C18—C17—C16	119.4 (4)
N5—Cd1—N1	79.74 (9)	C18—C17—H17	120.3
C1—N1—C5	118.7 (3)	C16—C17—H17	120.3
C1—N1—Cd1	111.40 (18)	C17—C18—C19	119.4 (4)
C5—N1—Cd1	121.4 (2)	C17—C18—H18	120.3
C10—N2—C6	119.4 (3)	C19—C18—H18	120.3
C10—N2—Cd1	123.3 (2)	C20—C19—C18	118.1 (3)
C6—N2—Cd1	116.9 (2)	C20—C19—H19	120.9
C15—N3—C11	119.1 (3)	C18—C19—H19	120.9
C15—N3—Cd1	123.0 (2)	N4—C20—C19	123.1 (3)
C11—N3—Cd1	116.1 (2)	N4—C20—H20	118.4
C20—N4—C16	119.1 (3)	C19—C20—H20	118.4
C20—N4—Cd1	123.3 (2)	N5—C21—C22	121.2 (4)
C16—N4—Cd1	117.5 (2)	N5—C21—C26	116.2 (3)
C25—N5—C21	118.0 (3)	C22—C21—C26	122.5 (3)
C25—N5—Cd1	122.9 (2)	C23—C22—C21	120.0 (4)
C21—N5—Cd1	114.7 (2)	C23—C22—H22	120.0
C30—N6—C26	119.6 (3)	C21—C22—H22	120.0
C30—N6—Cd1	122.2 (2)	C24—C23—C22	118.9 (4)
C26—N6—Cd1	117.6 (2)	C24—C23—H23	120.5
N1—C1—C2	121.6 (3)	C22—C23—H23	120.5
N1—C1—C6	116.5 (3)	C23—C24—C25	118.6 (4)
C2—C1—C6	121.8 (3)	C23—C24—H24	120.7
C3—C2—C1	119.0 (3)	C25—C24—H24	120.7
C3—C2—H2	120.5	N5—C25—C24	123.2 (4)
C1—C2—H2	120.5	N5—C25—H25	118.4
C4—C3—C2	119.0 (3)	C24—C25—H25	118.4
C4—C3—H3	120.5	N6—C26—C27	120.6 (4)
C2—C3—H3	120.5	N6—C26—C21	117.1 (3)
C3—C4—C5	119.4 (3)	C27—C26—C21	122.2 (3)
C3—C4—H4	120.3	C28—C27—C26	119.5 (4)
C5—C4—H4	120.3	C28—C27—H27	120.2
N1—C5—C4	122.2 (3)	C26—C27—H27	120.2
N1—C5—H5	118.9	C27—C28—C29	119.0 (4)
C4—C5—H5	118.9	C27—C28—H28	120.5
N2—C6—C7	120.9 (3)	C29—C28—H28	120.5
N2—C6—C1	116.6 (3)	C30—C29—C28	119.1 (4)
C7—C6—C1	122.5 (3)	C30—C29—H29	120.4
C8—C7—C6	119.4 (3)	C28—C29—H29	120.4
C8—C7—H7	120.3	N6—C30—C29	122.2 (4)
C6—C7—H7	120.3	N6—C30—H30	118.9
C7—C8—C9	119.2 (3)	C29—C30—H30	118.9
C7—C8—H8	120.4	O3—Cl1—O4	110.3 (3)
C9—C8—H8	120.4	O3—Cl1—O2	110.4 (2)
C10—C9—C8	118.1 (3)	O4—Cl1—O2	108.31 (19)
C10—C9—H9	120.9	O3—Cl1—O1	109.2 (2)
C8—C9—H9	120.9	O4—Cl1—O1	108.11 (19)
N2—C10—C9	123.0 (3)	O2—Cl1—O1	110.55 (16)
N2—C10—H10	118.5	O6—Cl2—O5	110.51 (17)
C9—C10—H10	118.5	O6—Cl2—O8	109.87 (19)
N3—C11—C12	120.6 (3)	O5—Cl2—O8	109.15 (16)
N3—C11—C16	116.4 (3)	O6—Cl2—O7	108.97 (17)
C12—C11—C16	123.0 (3)	O5—Cl2—O7	109.12 (16)
C13—C12—C11	120.0 (3)	O8—Cl2—O7	109.20 (17)
C13—C12—H12	120.0	H9A—O9—H9B	108.6
